# Mollusk shells as marine bioactive materials: Composition, bioactivities, and prospects for food and health applications

**DOI:** 10.1016/j.isci.2026.114748

**Published:** 2026-01-20

**Authors:** Jie-Ya Hou, Xian-Jun Fu, Xia Ren

**Affiliations:** 1 Research Institute for Marine Traditional Chinese Medicine (Qingdao Academy of Chinese Medical Sciences), The SATCM’s Key Unit of Discovering and Developing New Marine TCM Drugs, Key Laboratory of Marine Traditional Chinese Medicine in Shandong Universities, Shandong University of Traditional Chinese Medicine, Jinan 250355, China; 2 Shandong University of Traditional Chinese Medicine Qingdao Academy of Chinese Medical Sciences, Qingdao Key Laboratory of Research in Marine Traditional Chinese Medicine, Qingdao Key Technology Innovation Center of Marine Traditional Chinese Medicine’s Deep Development and Industrialization, Qingdao 266114, China

**Keywords:** Natural sciences, Biological sciences, Biochemistry, Biomaterials

## Abstract

Marine mollusk processing generates large quantities of shells that serve as abundant but underutilized biofunctional materials. Composed mainly of calcium carbonate with a minor organic matrix of proteins, polysaccharides, and chitin, mollusk shells exhibit characteristic activities such as antioxidant, anti-inflammatory, antimicrobial, osteogenic, hepatoprotective, gastrointestinal protective, and neuroprotective effects. These functions support their potential use as natural calcium sources, food preservation agents, functional ingredients, and biocompatible materials for tissue engineering and drug delivery. Safety concerns-particularly heavy metals and microplastics-are critically assessed alongside current mitigation approaches. Overall, current evidence supports MMSs as promising marine biomaterials with broad food and health applications. Persistent challenges include limited mechanistic understanding, species-dependent variability, and the lack of standardized processing and toxicological frameworks. Addressing these gaps will enable the safe and sustainable utilization of mollusk shells as high-value marine biomaterials.

## Graphical abstract

**Figure:**
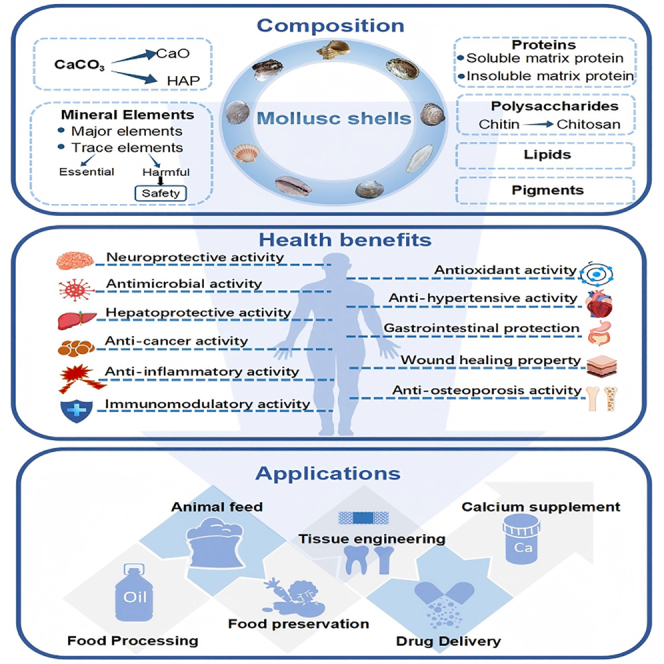

## Introduction

**Figure 1:**
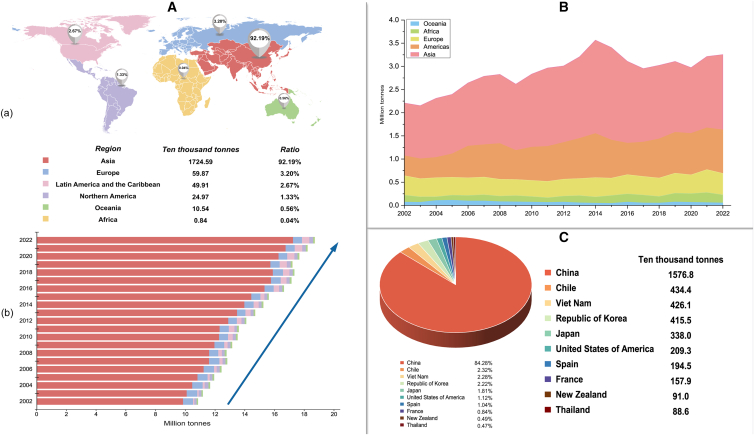
World overview of marine and coastal mollusks Aquaculture and Processed Production (2002–2022) (A) (a) World marine and coastal aquaculture of mollusks by region and ratio (2022). (b) World marine and coastal aquaculture of mollusks by region (2002–2022). (B) World mollusks Processed Production trends (2002–2022). (C) World marine and coastal aquaculture of mollusks by significant producers and ratio (2022). Source: FAO.2024. In: FishStatJ.

Aquatic animal food, encompassing fish, crustaceans, mollusks, and other invertebrates, represents a vital global food source with significant potential to improve food security and nutrition.^1^ Mollusks, in particular, are widely favored by consumers for their diversity and nutritional benefits. Bivalves, cephalopods, and gastropods are the most common and economically valuable categories of mollusks.^2^ According to the 2024 edition of *The State of World Fisheries and Aquaculture*,^3^ mollusk farming and processing have seen a steady rise in recent years. In 2022, the global production of marine and coastal aquaculture reached 35.34 million tons of aquatic animals. As a major species group, mollusks accounted for 52.9% of total aquatic animal production, reaching 18.7 million tons, with significant regional differences. Asian countries accounted for 92.19% of the global marine and coastal mollusk aquaculture production, followed by Europe (3.20%), Latin America and the Caribbean (2.67%), North America (1.33%), Oceania (0.56%), and Africa (0.04%) (Figure 1).

Processing plays a critical role in the production chain of aquatic animals from ocean to table.^4^ Most species of marine mollusks have non-edible shells, which constitute 60–80% of their total live weight and serve as the primary byproduct of the processing industry.^5^ These marine mollusk shells (MMSs) present both significant environmental challenges and opportunities for value-added applications. Due to current limitations in shell disposal technologies, these byproducts are frequently discarded into the ocean or piled up on beaches for landfilling, resulting in soil, air, and water contamination and causing significant damage to marine ecosystems and the environment.^6^ Therefore, identifying cost-effective and environmentally friendly methods to recover and repurpose these MMSs is a critical issue for ensuring the sustainable development of the marine mollusk aquaculture industry.

Historically, mollusk shells have been used worldwide for medicinal, cosmetic, and therapeutic applications. Records from the ancient Chinese medical monographs *Huangdi Neijing* describe the use of cuttlebone in formulations for treating blood depletion disorders over 2,000 years ago.^7^ During the 14th century, powdered cuttlebone was applied in Spain for skin whitening and dental cleaning,^8^ and by the 18th century, mussel shells were used in Latin America for treating ocular conditions.^9^ Moreover, mollusk shells across different regions were often processed in similar ways and for similar purposes. Shells in the natural or calcined state were typically prepared in powdered form or dissolved in various solvents, including water and vinegar, and used as treatments for ailments such as gastric ulcers, as wound-healing agents, and as sedatives. Today, several types of MMSs are still used in clinical treatments in China; the 2025 edition of *the Pharmacopoeia of the People’s Republic of China* includes six types of MMSs: *Arcae Concha*, *Haliotidis Concha*, *Margaritifera Concha*, *Meretricis Concha*, *Ostreae Concha*, and *Sepiae Endoconcha*.^10^

The exploration of mollusk shells as bioactive materials has also evolved considerably in scientific research. Early investigations in the mid-20th century largely focused on mineralogical composition and shell architecture, laying foundational knowledge of their hierarchical structure and mechanical performance.^11^ From the 1990s onward, advances in biomineralization research revealed the presence of complex organic matrices-proteins, peptides, and polysaccharides-embedded within the mineral phase, shifting attention toward their biochemical and functional roles.^12^ In the past two decades, research has expanded from fundamental characterization to application-oriented studies in osteogenesis, wound healing, environmental remediation, and, most recently, drug delivery.^13^ These developments trace a transition from traditional empirical uses to mechanistic understanding and modern translational potential.

Despite their widespread availability and rich biofunctional potential, MMSs have received far less scientific attention than crustacean shells. Existing reviews have primarily focused on crustacean-derived chitin, chitosan, and mineral materials, with limited emphasis on mollusk shells.^14^^,^^15^ Moreover, current literature lacks an integrated and systematic overview of MMSs that encompasses their chemical composition, biological activities, food-related applications, and safety considerations. The lack of an integrated and comprehensive summary has constrained the systematic understanding and further development of MMS-derived materials, particularly in relation to their potential applications in the food, health, and biotechnology sectors.

To address this gap, the present review provides a consolidated and up-to-date synthesis of the composition, extraction strategies, biological properties, and emerging applications of MMSs, with an emphasis on their functional and health-promoting potential. Particular attention is given to food science-related applications, bioactive properties, and safety considerations that are essential for ensuring the sustainable and reliable use of MMSs. This review aims to highlight the unique value of MMSs and provide a foundation for their broader utilization as sustainable biomaterials in food and health industries.

## Composition and structure of marine mollusk shells

**Figure 2:**
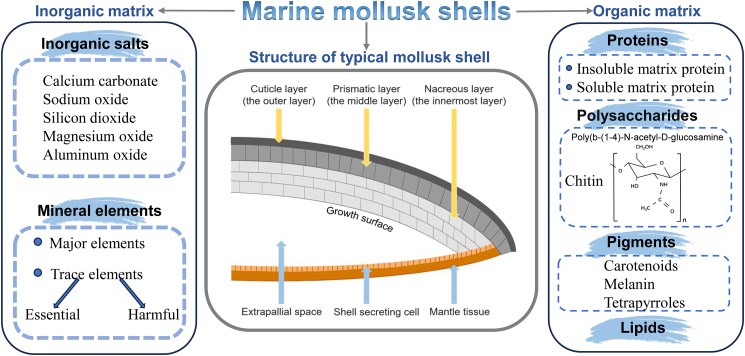
Composition and structure of marine mollusk shells (MMSs)

MMSs are organo-mineral biocomposites secreted by the mantle epithelium and consist of approximately 95% inorganic components, mainly calcium carbonate, and 1–5% organic materials, including proteins, chitin, lipids, and pigments.^6^ MMSs are typically composed of three distinct layers: the cuticle, the prismatic layer, and the nacre layer (Figure 2).^16^

In terms of structure, the cuticle, forming the outermost layer, is primarily composed of hardened proteins that offer protection and prevent shell dissolution.^17^ The prismatic layer, located in the middle, consists of aragonite or calcite crystals arranged in parallel, providing the shell with enhanced hardness and corrosion resistance.^18^ The innermost nacre layer is characterized by alternating layers of aragonite crystals and organic matrices in a “brick-and-mortar” structure, imparting the shell with remarkable toughness and strength.^19^ While microscopic structural differences exist among species, all mollusk shells demonstrate exceptional mechanical properties.

In terms of composition, MMSs are composed of both inorganic and organic matrices. Calcium carbonate is the predominant inorganic component, along with other inorganic salts (e.g., sodium oxide, silicon dioxide, magnesium oxide, and aluminum oxide) and mineral elements.^20^ The major elements found in MMSs include calcium (Ca), magnesium (Mg), sodium (Na), chloride (Cl), sulfur (S), phosphorus (P), and potassium (K). Trace elements such as iron (Fe), copper (Cu), zinc (Zn), chromium (Cr), nickel (Ni), strontium (Sr), manganese (Mn), vanadium (V), bromine (Br), molybdenum (Mo), selenium (Se), and cobalt (Co) are also present. Essential trace elements are critical for human metabolism and immune function, whereas harmful trace elements, including lead (Pb), cadmium (Cd), arsenic (As), and mercury (Hg), raise safety concerns in practical applications.^21^ Although the exact mineral composition varies substantially across species, published studies provide indicative ranges for major and trace elements. Magnesium typically accounts for 0.2–1.2% of shell dry weight, sodium 0.1–0.8%, and phosphorus 0.05–0.3%, while trace elements such as iron, copper, zinc, strontium, and manganese generally appear at concentrations below 0.01–0.1% of dry weight. These values differ widely depending on species, geographic origin, environmental conditions, and analytical methods employed. Essential trace elements contribute to normal physiological functions, whereas potentially toxic elements may occur at μg/kg to mg/kg levels, underscoring the need for rigorous safety assessment.

The organic matrix is a complex mixture of proteins, polysaccharides, lipids, and pigments. Although comprehensive quantitative data remain scarce, existing studies report that proteins constitute approximately 0.1–2% of shell dry weight, whereas chitin and polysaccharides collectively account for 0.1–1%, depending on species and layer type.^20^ Lipids and pigments are present only in trace amounts. Due to the extremely low abundance of organic components and the challenges associated with separating tightly bound insoluble matrix proteins, systematic quantification across species remains limited. Shell matrix proteins, crucial in guiding and regulating biomineralization, are generally classified into soluble and insoluble matrix proteins (IMPs). IMPs are tightly bound to chitin, presenting challenges for studying these components, while soluble matrix proteins can be isolated and identified using high-throughput techniques such as proteomics, genomics, and transcriptomics.^22^ Polysaccharides, particularly chitin-a biopolymer of β-1,4-N-acetylglucosamine-are a significant part of the organic matrix and are widely present in shells.^23^ Farre, & Dauphin.^24^ confirmed the presence of lipids in MMSs by Fourier transform infrared spectroscopy, with notable species-dependent variations. In a study by Williams,^25^ pigments such as carotenoids, melanins, and tetrapyrroles (including porphyrins and bile pigments) were identified as the primary pigments found in MMSs.

## Recovery of functional components from marine mollusk shells

**Figure 3:**
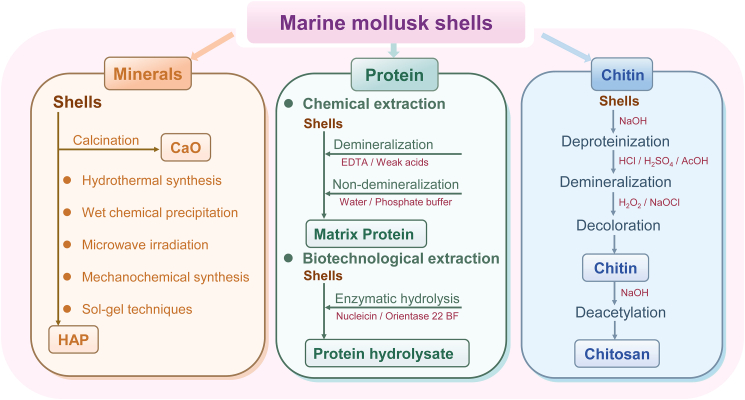
Recovery of functional components from marine mollusk shells (MMSs)

MMSs are a valuable source of bioactive compounds. Currently, the recovery of functional components from MMSs has become a major focus in the field of high-value shell utilization, with particular attention given to the recovery of minerals, proteins, and chitin. Figure 3 provides an overview of commonly used extraction techniques for recovering functional components from MMSs, including basic processes and commonly used reagents, along with the products obtained, to facilitate the reutilization of MMSs.

### Minerals

MMSs, as a renewable source of calcium carbonate, serve as precursors for producing calcium oxide (CaO) and hydroxyapatite (Ca_10_(PO_4_)_6_(OH)_2_, HAP). Through calcination, the calcium carbonate in MMSs is converted into calcium oxide, with the optimal calcination temperature varying by shell type.^26^ During this process, both the composition and structure of the shells undergo significant changes. The aragonite phase of calcium carbonate is usually converted into the more stable calcite phase, while the protein content markedly decreases, and the levels of major elements such as Ca, Na, and P increase.^20^ MMSs-derived CaO has shown considerable potential in applications such as antimicrobial agents and antacids. Furthermore, there has been notable progress in synthesizing HAP using MMSs as a natural calcium source, in combination with phosphate precursors such as (NH_4_)_2_HPO_4_ and H_3_PO_4_.

Several categories of synthetic methods have been developed, which can be broadly classified into solution-based, energy-assisted, and solid-state methods, each with unique advantages and limitations.^6^ Solution-based methods, including hydrothermal synthesis and wet chemical precipitation, remain the most widely applied. Hydrothermal methods utilize elevated temperature and pressure to promote controlled crystal growth, enabling fine-tuning of particle size and morphology.^27^ Wet precipitation, by adjusting pH, temperature, and ion concentrations, allows efficient nucleation and yields uniform HAP powders, although it requires careful control to avoid agglomeration.^28^ Energy-assisted approaches, such as microwave irradiation, accelerate reaction kinetics through rapid volumetric heating, producing nanoscale HAP with narrow size distributions and reduced synthesis time.^29^ Solid-state methods, represented by mechanochemical synthesis and sol-gel processing, minimize solvent use and environmental impact. Mechanochemical milling promotes solid-state reactions between calcium and phosphate precursors,^30^ whereas sol-gel techniques produce highly homogeneous gels that convert into HAP with tunable porosity after drying and calcination.^31^

These synthesis strategies clearly demonstrate the versatility of MMSs as a sustainable calcium source but also underscore several technical challenges. Natural variation in mineral composition among mollusk species, batch-to-batch inconsistencies in shell impurities, and the presence of residual organic matter can influence nucleation kinetics, particle size uniformity, and final crystallinity, thereby affecting the reproducibility and performance of MMS-derived materials. In addition to their structural stability, ion-exchange capacity, and strong adsorption properties, MMSs-derived HAP typically contains naturally occurring trace elements such as Mg, Sr, Si, Zn, and K. These ions are known to enhance osteogenic differentiation, cell adhesion, proliferation, and overall bioactivity, further improving the suitability of MMSs-derived HAP for bone tissue engineering, drug delivery, and broader biomedical applications.^32^ However, the incorporation of trace elements also introduces challenges related to compositional control, safety evaluation, and standardization. These considerations highlight the need for more rigorous purification procedures and comprehensive physicochemical characterization to ensure consistent quality and safe implementation of MMSs-derived mineral materials.

### Proteins

Proteins, as vital organic components of MMSs, have attracted significant attention for their critical role in shell biomineralization. Recent studies indicate that soluble matrix proteins derived from MMSs exhibit notable bioactivity, positioning them as promising candidates for functional food and pharmaceutical applications. The extraction of these matrix proteins typically involves both chemical and biotechnological techniques, often paired with chromatographic separation for purification. Chemical extraction methods can follow either demineralization or non-demineralization processes. Demineralization uses reagents such as ethylenediaminetetraacetic acid (EDTA) or weak acids for protein extraction and offers the advantage of high extraction efficiency,^33^^,^^34^ though it may result in the denaturation or degradation of certain proteins due to prolonged exposure to acidic or chelating environments. Non-demineralization methods, considered gentler, employ non-toxic reagents such as water or phosphate buffer, preserving the integrity and bioactivity of the proteins.^35^^,^^36^

Biotechnological extraction techniques, particularly enzymatic hydrolysis, have gained increasing attention for producing bioactive protein fragments under mild and controllable conditions. For example, Sasaki et al.^37^ employed the proteases Nucleicin and Orientase 22 BF to hydrolyze proteins from pearl oyster shells, with Orientase achieving the highest degree of hydrolysis (26%) after 6 h. Factors such as reaction time, temperature, and enzyme-to-substrate ratios significantly influence hydrolysis efficiency and the resulting peptide composition.

Despite recent progress, the extraction and purification of MMS-derived proteins still face several challenges. Shell protein content is inherently low, and their tight association with the mineral matrix complicates separation. Species-dependent variability, heterogeneity of the organic matrix, susceptibility to denaturation during processing, and difficulty achieving consistent chromatographic purification further limit the scalability and reproducibility of current methods. Developing standardized extraction protocols and gentle, high-yield separation technologies remains essential for advancing the use of MMS proteins in functional and biomedical applications.

Collectively, these emerging extraction strategies have expanded the potential of MMS-derived proteins beyond their structural roles, supporting their growing relevance as promising bioactive ingredients for food, health, and pharmaceutical industries.

### Chitin

MMSs contain chitin, which can be converted into the biopolymer chitosan through a process of deacetylation. Conventional extraction generally involves four sequential steps: deproteinization, demineralization, decoloration, and deacetylation.^38^ Deproteinization typically employs alkaline treatment with sodium hydroxide (NaOH) to break the chemical bonds between proteins and chitin. The concentration of NaOH, along with the treatment time and temperature, varies depending on the type of shell being processed.^39^ Demineralization is then carried out using acids such as hydrochloric acid (HCl), sulfuric acid (H_2_SO_4_), or acetic acid (HOAc), with HCl being the most widely used.^40^ To obtain a colorless product, oxidizing agents, including hydrogen peroxide or sodium hypochlorite, are applied, followed by further alkaline treatment to deacetylate chitin into chitosan. The resulting chitosan is hydrophilic, biodegradable, and biocompatible, making MMS-derived chitosan suitable for food packaging, preservation, and biomedical applications.^41^

With increasing interest in sustainable extraction technologies, Deep eutectic solvents (DESs) are emerging as green alternatives to harsh acids/alkalis for chitin isolation because of their negligible vapor pressure, biodegradability, facile recyclability, and structural mildness toward polysaccharides.^42^ McReynolds et al.^43^ used European squid *(Loligo vulgaris) pens* as feedstock and systematically evaluated six acidic, neutral, and alkaline deep eutectic solvents (DESs) for β-chitin extraction. Only the alkaline K_2_CO_3_–glycerol (KGLY) system at 100°C–120°C for 2–3 h delivered high-purity, highly crystalline β-chitin comparable to the conventional alkaline route. The solvent retained ∼30% yield and constant viscosity over three consecutive cycles without additional demineralization, offering a shorter, milder, recyclable, and environmentally benign protocol for upgrading low-mineral fishery by-products.

Despite these advances, the application of DES-based extraction to MMSs remains limited. To date, such processes have been deployed almost exclusively for chitin recovery from crustacean waste shells. Compared with crustaceans, mollusk shells feature distinct mineral compositions, higher degrees of calcification, and lower chitin content, presenting additional challenges for solvent penetration and mass transfer. Conventional acid-alkali methods currently address these challenges more directly, though at the expense of environmental burden, potential protein denaturation, and partial chitin degradation. DES-based processes represent a promising future direction for MMSs, offering the potential for higher-purity chitin and reduced chemical waste. However, further studies are required to tailor DES formulations to the unique physicochemical properties of MMSs, improve extraction efficiency, and enable greener, scalable production of mollusk-derived chitin.

## Biological properties of marine mollusk shells

MMSs and calcined MMSs have numerous health benefits *in vitro* and *in vivo*, such as antioxidant, anti-inflammatory, antibacterial, and anti-osteoporotic effects; hypertension regulation; and wound healing. Table 1 comprehensively summarizes the biological properties of MMSs.

**Table 1:** Biological properties of marine mollusk shells (MMSs) in literatures

Biological properties	Materials	Source	Experiment model	main finding	Reference
Antioxidant activity	Oyster shell 10% HCl solution	–	*in vitro* antioxidant assay	Scavenging activity of hydroxyl radical and superoxide radical	Ma^44^
Antioxidant activity	Ark shell Proteins: WLZP1F1G2, WLZP1F1G3	*Arca inflata*	*in vitro* antioxidant assay; *in vivo C. elegans.* Test	Scavenging activity of DPPH radical, ABTS radical; anti-aging capacity ↑	Zheng et al.^45^
Antioxidant activity	abalone shell water extract	–	H_2_O_2_ induced mouse lens/human lens epithelial cells model	LDH, HLEC, MD ↓; GSH ↑, SOD activity ↑	Cui & Xu,^46^ Zhou et al.^47^
Antioxidant activity	Pearl oyster shell Water extract	*Pinctada maxima*	D-Galactose induced ICR mice model	MDA ↓; GSH ↑, GPx, CAT, SOD activity ↑	Yamamoto et al.^45^
Anti-osteoporosis activity	Oyster shell water-soluble matrix protein (WSMP)	*Crassostrea gigas*	mouse preosteoblast MC3T3-E1 cell line	ALP, OCN, BMP-2 ↑, Cell proliferation rate↑	Feng et al.^35^^,^^48^
Anti-osteoporosis	Oyster shell WSMP	*Crassostrea gigas*	Retinoic acid-induced osteoporosis in the SD rat model	ALP, BMP-2↑ BMD, BMC↑	Feng et al.^35^^,^^48^
Anti-osteoporosis	Pearl oyster shell Protein:N16	*Pinctada martensi*	GIO rat model	BMD ↑; inducing osteoblast biomineralization, inhibiting osteoclast formation	Xu et al.^49^
Anti-osteoporosis	Pearl oyster shell Protein:N16	*Pinctada martensi*	prednisolone-induced larval zebrafish (*Danio rerio*) model	osteoblastic characteristic genes ↑ osteoclastic characteristic genes ↓	Lin et al.^50^
Anti-osteoporosis	Pearl oyster shell powder	*Pinctada maxima*	Ovariectomized female Wistar rats model	CTX ↓, P1NP ↑	Nguyen et al.^51^
Anti-osteoporosis	Calcined oyster shell powder	–	GIO rat model	bone mineral loss ↓ serum calcium levels ↓ bone mineral density and trabecular thickness ↑	Shi et al.^52^
Anti-hypertensive	Oyster shell Water extract	*Ostrea rivularis Gould*	Fuzi tang induced-SHR rat hypertension of the liver-yang model	blood pressure ↓; NE, E, Ang Ⅱ, ALD ↓; NO ↑	Sheng et al.^53^
Anti-hypertensive	abalone shell Water extract	*Haliotis diversicolor Reeve*			
Anti-hypertensive	ark shell Water extract	*Arca subcrenata Lischke*			
Anti-hypertensive	Pearl oyster shell Peptide: Gly-Val-Gly-Ser-Pro-Tyr	*Pinctada fucata*	*In vitro* HHL assay	ACE ↓, IC50: 5.82 μg/mL	Sasaki et al.^37^
Anti-hypertensive	abalone shell Water extract	*Haliotis discus hannai Ino*	*In vitro* HHL assay	ACE ↓, IC50: 49.20 mg/mL	Ma et al.^54^
Anti-hypertensive	abalone shell Water extract	–	SHR rat model	blood pressure ↓	Chen et al.^55^
Anti-inflammatory activity	Oyster shell water extract	–	LPS-treated RAW 246.7 cells	IL-1β, IL-6, TNF-α, NO, NF-κB, iNOS, COX-2 ↓	Lee et al.^56^
Immunomodulatory activity	ark shell Proteins:WLZP-1,WLZP-2	–	RAW 246.7 cells	NO ↑	Shi et al.^57^
Anti-cancer activity	Oyster shell methanol extract	–	K562,HL-60,Hela,A549,BEL-7402,SMMC-7721,OS-RC-2,CaO-2,MGC90-3 cells	Cell proliferation ability ↓	Yang et al.^58^
Anti-cancer activity	Oyster shell Powder, Oyster shell calcium	*Ostrea gigas*	4NQO-fed C57BL/6 mice	The formation and proliferation of oral squamous cell carcinoma ↓, PCNA ↓ keratin 1, involucrin, filaggrin, and loricrin ↑	Chen et al.^59^
Wound healing	Abalone shell Powder solution	*Haliotis diversicolor*	LSP-induced RAW 264.7	Cell proliferation ↑, iNOS ↓	Chen et al.^60^
Wound healing	Abalone shell Powder solution	*Haliotis diversicolor*	Wistar rat burn injury model	TGF-β1 ↑; type I collagen ↑	Chen et al.^60^
Wound healing	Pearl shell water-soluble factor	*Pteria martensii*	murine fibroblast NIH3T3cells	Cell proliferation ↑; type I collagen ↑	Lee et al.^61^
Wound healing	Pearl shell water-soluble factor	*Pteria martensii*	*Sus scrofa domesticus* female porcine second-degree burn model	collagen deposition ↑; necrotic tissue clearance ↑, granulation tissue formation ↑	Lee et al.^61^
Wound healing	Cuttlebone water extract	*Sepia esculenta Hoyle*	*In vitro* PT、APPT assay	Coagulation time ↓; PT、APPT ↓	Liu et al.^62^
Gastrointestinal protective properties	Cuttlebone water extract	*Sepiella maindroni de Rochebrune*	Indomethacin-induced gastric mucosal lesions in the rat model	NO, PEG2, SOD, EGF ↑; MDA ↓	Qiu et al.^63^
Gastrointestinal protective properties	Oyster shell water extract; Calcined -shell water extract	*Ostrea gigas Thunberg*	Anhydrous ethanol-induced acute gastric ulcer rat model	Gastric juice secretion ↓, PH ↓, Pepsin activity ↓	Nie et al.^64^
Gastrointestinal protective properties	ark shell water extract; Calcined -shell water extract	*Arca subcrenata*	Anhydrous ethanol-induced acute gastric ulcer rat model	NO, PEG2, SOD, EGF ↑; MDA ↓	Fang et al.^65^
Gastrointestinal protective properties	Cuttlebone Polysaccharide:CPS-1	*Sepiella maindroni de Rochebrune*	DSS-induced UC mice model	EGF, PDGF ↑; TNF-α ↓	Wei et al.^66^
Hepatoprotective activity	Oyster shell Ethanol extract	Crassaostrea gigas	murine Hepa1c1c7 hepatoma cells	Sptlc1,Sptlc2 ↓; SPA, SM ↓, ACC, FAS, SCD1, DGAT2 ↓, TG ↓	Tran et al.^67^
Hepatoprotective activity	abalone shell water extract	*Haliotis discus hannai Ino, Haliotis ruber(Leach), Haliotis laevigata (Donovan)*	CCl_4_-induced hepatotoxicity Swiss mouse model	Glycogen ↑; alleviates inflammation in the portal area and promotes hepatocyte regeneration.	Li et al.^68^
Hepatoprotective activity	Cuttlebone chitosan	*Sepia kobiensis*	CCl_4_-induced hepatotoxicity in the Wistar rat model	MDA↓; GSH↑, SOD, CAT, GPx↑; ALT, AST, FFA, TG, TC, HDL-C↓	Ramasamy et al.^69^
Neuroprotective	Pearl oyster shell water extract	*Pinctada maxima*	LPS-induced depression and anxiety in mice model	Nrf2↑; 5-HT1A↑, 5-HT2A↓; BDNF↑	Omachi et al.^70^
Neuroprotective	Pearl oyster shell water extract	*Pinctada fucata*	Scopolamine-Induced ICR mice Memory Impairment model	MDA↓, Cu, Zn-SOD↑; IL-1β, IL-6, TNF-α↓; BDNF, NGF↑	Yamagami et al.^71^
Neuroprotective	Pearl oyster shell powder, water-soluble protein, conchiolin protein	–	PTZ-induced convulsion mice model	5-HT3↓, GABA↑	Zhang et al.^72^

### Antioxidant activity

Oxidative stress is closely associated with the onset of various diseases, including eye disorders, neurological conditions, cancer, and cardiovascular diseases. Numerous studies have highlighted that MMSs demonstrate significant antioxidant properties, which may offer health benefits in combating oxidative damage.

*In vitro*, the antioxidant activity of the oyster shell was evaluated using superoxide radical- and hydroxyl radical-scavenging assays. The glycoprotein LOL extracted from oyster shells with 10% HCl showed hydroxyl radical (43.72%) and superoxide radical (5.18%) scavenging capacities. Notably, the ·OH-scavenging capacity of this glycoprotein surpassed that of vitamin C (35.8%).^44^ Additionally, a combination of sonication-assisted water decoction extraction and column chromatography was used to extract WLZP1F1G2 and WLZP1F1G3 proteins from ark (Arca inflata) shells. Their radical scavenging capacities were evaluated through 2,2-azinobis-(3-ethyl-benzthiazoline-6-sulfonate) (ABTS) and 1,1-diphenyl-2-picrylhydrazyl (DPPH) assays. The half-maximal effective concentration (EC50) values for WLZP1F1G2 were 1167.22 μg/mL (ABTS) and 1321.77 μg/mL (DPPH), while for WLZP1F1G3, they were 576.35 μg/mL (ABTS) and 356.67 μg/mL (DPPH), indicating significant radical scavenging potential for both proteins.^45^ In another study, Cui & Xu^46^ and Zhou et al.^47^ explored the effects of abalone shell extracts on oxidative stress. Mouse lens and human lens epithelial cells (HLECs) were cultured *in vitro*, and oxidative stress damage was induced by hydrogen peroxide (H_2_O_2_); pretreatment with abalone shell water extract alleviated H_2_O_2_-induced lens opacity and also mitigated the reduction in cellular proliferation and viability caused by oxidative stress. Furthermore, it reduced the release of lactate dehydrogenase (LDH) from the lenses and diminished the accumulation of malondialdehyde (MDA) within cells (HLECs) while enhancing glutathione (GSH) content and superoxide dismutase (SOD) activity.

The antioxidant effects of MMSs have also been confirmed *in vivo*. *Caenorhabditis elegans* was cultured in a medium containing ark shell protein, and various aging markers were assessed. The results indicated that ark shell protein extended the lifespan of *C. elegans*; significantly reduced the accumulation of fat, lipofuscin, and reactive oxygen species (ROS); and showed promising anti-aging effects.^45^ In mouse brain tissue, D-Galactose induces oxidative stress, increasing MDA levels and the expression of the cellular senescence marker p16. Treatment with pearl oyster (Pinctada maxima) shell water extract, however, enhanced GSH content and glutathione peroxidase (GPx) activity, upregulated the expression of catalase (CAT) and SOD, inhibited the rise in MDA levels and p16 expression, and boosted free radical-scavenging and iron-reducing capacity, thereby mitigating brain aging.^73^

Given these findings, MMSs hold promise as key components in functional foods and antioxidant formulations, offering potential health benefits in mitigating oxidative damage.

### Antimicrobial activity

Antibacterial activity has long been a hotspot in MMS research. Studies have shown that MMSs are a promising source of natural antibacterial agents with potential applications in extending food shelf life and preventing foodborne diseases. Shells in their natural state, calcined shells, and organic matrix extracts of MMSs exhibit varying degrees of antibacterial and antifungal activity, which are influenced by factors such as the bacterial strain, shell type, extraction solvent, and heat treatment temperature.^74^ Among these, organic matrix extracts showed the lowest antibacterial activity, while calcined MMSs exhibited the highest antibacterial activity, displaying potent and broad-spectrum activity against both bacteria and fungi.

The high antibacterial activity of calcined MMSs is likely related to their primary component, CaO. The primary component of natural shells, CaCO_3_, undergoes thermal decomposition during heat treatment, forming CaO. CaO creates a highly alkaline environment that damages bacterial cell walls, leading to microbial death. This mechanism is especially effective against Gram-positive bacteria, which are more susceptible to cell wall penetration due to structural differences compared to Gram-negative bacteria.^5^ In antibacterial studies of calcined MMSs, Cui et al.^75^ observed that, within a certain temperature range, the inhibitory capacity of MMSs against tested bacteria significantly increases with higher heat treatment temperatures, and the antibacterial effect is positively correlated with the concentration.

Additional quantitative evidence further confirms the dose-dependent and species-specific antimicrobial effects of calcined MMSs. Hu et al.^76^ evaluated calcined powders of mussel, *Meretrix meretrix*, and *Sinonovacula constricta* shells at concentrations of 1%, 0.1%, and 0.01% (w/v) against four major foodborne pathogens—*Salmonella enterica*, *Escherichia coli*, *Vibrio parahaemolyticus*, and *Staphylococcus aureus*. At a concentration of 1%, all calcined MMS powders exhibited bactericidal activity. Notably, calcined *S. constricta* and *M. meretrix* shells reduced *V. parahaemolyticus* to below 10 CFU/mL within 5 min, and eliminated *E. coli* to undetectable levels within 10 min. Under the concentration of 0.1 g/mL, the inhibitory effect of *S. constricta* calcined shell powder on Salmonella was the strongest, and the diameter of the inhibition zone was 16.42 mm. These findings provide strong experimental validation for the rapid and broad-spectrum antimicrobial potential of calcined MMSs.

### Anti-osteoporosis activity

In recent years, MMSs have gained considerable attention for their potential role in preventing and treating osteoporosis. Research suggests that MMSs exhibit anti-osteoporotic properties by stimulating bone formation and suppressing bone resorption.

Water-soluble matrix proteins (WSMPs) extracted from oyster (*Crassostrea gigas*) shells have demonstrated significant efficacy in both *in vitro* and *in vivo*, promoting the proliferation, differentiation, and mineralization of osteoblasts while inhibiting the resorptive activity of osteoclasts.^35^^,^^48^ *In vitro*, WSMPs suppress the differentiation of RAW264.7 cells into osteoclasts in response to nuclear factor-kappa B (NF-κB) ligand (RANKL) and significantly enhance the proliferation of the mouse preosteoblast cell line MC3T3-E1. Additionally, WSMPs upregulate the expression of alkaline phosphatase (ALP), osteocalcin (OCN), and bone morphogenetic protein (BMP)-2 in differentiated osteoblasts, leading to the increased formation of mineralized nodules. *In vivo*, WSMPs have been shown to increase bone mineral density (BMD) and bone mineral content (BMC) in retinoic acid-induced osteoporotic rats while also modulating osteoclast differentiation pathways.

Similarly, the active protein N16, isolated from pearl oyster (*Pinctada martensi*) shell, demonstrates dual regulatory effects on both osteoblasts and osteoclasts. N16 has shown therapeutic potential in osteoporosis models, such as dexamethasone-induced osteoporosis in female rats and prednisolone-induced osteoporosis in larval zebrafish (*Danio rerio*).^49^^,^^50^ Its mechanism involves the upregulation of osteoblastic gene expression and the downregulation of osteoclastic gene expression.

Moreover, incorporating pearl oyster (*Pinctada maxima*) shell as a dietary calcium supplement has been shown to mitigate estrogen deficiency-induced bone loss in ovariectomized (OVX) rats by correcting imbalances in bone turnover, Compared to standard CaCO_3_ supplementation, nacre supplementation attenuated the rise in serum levels of C-terminal collagen cross-linking telopeptide of type I collagen (CTX) and increased the secretion of procollagen type I N-terminal propeptide (P1NP).^51^ Similarly, calcined oyster shell powder supplementation in glucocorticoid-induced osteoporosis (GIO) mouse models was found to prevent bone mineral loss, improve BMD and trabecular thickness, enhance bone microarchitecture, reduce serum calcium levels, and positively influence the gut microbiota linked to bone health.^52^

### Anti-hypertensive activity

Hypertension is a chronic condition characterized by persistently elevated arterial blood pressure, often leading to organ damage, including the heart, brain, and kidneys. MMSs have demonstrated promising anti-hypertensive effects through multiple regulatory pathways.

Spontaneously hypertensive rats (SHRs) were administered Fuzi Decoction by gavage to establish a model of hypertension of the Liver-yang hyperactivity type. Following treatment with water extracts of oyster (*Ostrea rivularis Gould*) shell, abalone (*Haliotis diversicolor Reeve*) shell, and ark (*Arca subcrenata Lischke*) shell, the systolic blood pressure significantly decreased, and the rats' irritability, facial temperature, and conjunctival congestion were notably improved. Levels of norepinephrine (NE), epinephrine (E), angiotensin II (AngII), and aldosterone (ALD) in plasma were reduced, while plasma NO levels increased. The potential mechanism may involve the regulation of catecholamine (CA) levels, blockade of the renin-angiotensin-aldosterone system (RAAS), and inhibition of vascular remodeling.^53^

Angiotensin-converting enzyme (ACE) is an important regulatory factor in the RAAS. *In vitro*, both hydrolyzed proteins from pearl oyster (*Pinctada fucata*) shell and aqueous extracts of abalone shell exhibit significant inhibitory effects on ACE.^37^^,^^54^ The inhibitory activity is evaluated by measuring the production of hippuric acid from the reaction of hippuryl-his-leu hydrate (HLL) with ACE. The IC50 value for the aqueous abalone shell extract was found to be 49.2 mg/mL, while an ACE inhibitory peptide isolated from pearl oyster shell demonstrated potent activity with an IC50 of 5.82 μg/mL. The identified amino acid sequence of this peptide is Gly-Val-Gly-Ser-Pro-Tyr (MW: 578.7 Da).

Furthermore, abalone shell water extracts may reduce blood pressure by modulating calcium ion channels. In SHR, the intervention of abalone shell water extract decreased the expression of CaL-α1C mRNA, reduced ICa-L flow in vascular smooth muscle cells, increased serum calcium levels, and consequently lowered blood pressure.^55^

### Anti-inflammatory and immunomodulatory properties

Inflammatory responses and immune dysfunction are key factors in the development of many diseases. Research shows that oyster shell water extracts exert significant anti-inflammatory effects in lipopolysaccharide (LPS)-stimulated RAW 264.7 cells without inducing cytotoxicity.^56^ The extract inhibits the production of pro-inflammatory cytokines, including interleukin-1β (IL-1β), interleukin-6 (IL-6), and tumor necrosis factor-alpha (TNF-α), as well as the expression of inflammatory mediators such as nitric oxide (NO), nuclear factor-kappa B (NF-κB), inducible nitric oxide synthase (iNOS), and cyclooxygenase-2 (COX-2). Additionally, the extract reduces intracellular ROS levels and enhances antioxidant enzyme activity in a dose-dependent manner.

In another study, Shi et al.^57^ have highlighted the immunomodulatory effects of ark shell proteins. The water-soluble protein WLZP-1 and the acid-soluble protein WLZP-2 from ark shell significantly promote NO production in RAW 264.7 cells, demonstrating strong immuno-enhancing activity. WLZP-1, in particular, shows superior immuno-enhancing effects compared to WLZP-2. Proteomic and transcriptomic analyses have identified eight and seven protein components in WLZP-1 and WLZP-2, respectively, that are involved in human immune system regulation. These active proteins provide the molecular basis for the immunomodulatory properties of ark shell, underscoring its potential role in supporting immune function.

Importantly, inflammation and immune regulation are deeply interconnected processes that influence a broad range of physiological and pathological pathways. Chronic inflammation is now recognized as a critical hallmark of the tumor microenvironment (TME), shaping immune cell recruitment, cytokine signaling, oxidative stress, and tumor progression.^77^^,^^78^ Given that MMS-derived extracts can inhibit pro-inflammatory signaling (e.g., NF-κB and ROS) while activating macrophage-mediated immune functions, these dual activities may have downstream implications for modulating the TME. For example, the suppression of inflammatory cytokines may attenuate tumor-promoting inflammation, while immune-activating proteins may enhance antitumor macrophage functionality. Although direct evidence linking MMS components to TME regulation remains limited, these mechanistic connections highlight a promising area for future research and suggest potential synergy with their previously reported antitumor effects.

### Anti-cancer activity

MMSs exhibit notable anticancer and antitumor properties, both *in vitro* and *in vivo*. *In vitro*, using methanol extracts of oyster shells have demonstrated broad-spectrum activity against various tumor cell lines, including human chronic myeloid leukemia (K562), human promyelocytic leukemia (HL-60), human cervical cancer (HeLa), human non-small cell lung adenocarcinoma (A549), human liver cancer (BEL-7402, SMMC-7721), human renal carcinoma (OS-RC-2), human colorectal adenocarcinoma (CaO-2), and human gastric cancer (MGC90-3). These findings suggest that oyster shell extracts have considerable potential for inhibiting tumor cell proliferation across a range of cancer types.^58^

*In vivo*, oyster (*Ostrea gigas*) shell can prevent the development of oral cancer by suppressing the formation and proliferation of oral squamous cell carcinoma and promoting the differentiation of oral epithelial cells. For 16 consecutive weeks, C57BL/6 mice were fed with the carcinogen 4-nitroquinoline-1-oxide (4NQO, 50 μg/mL), and divided into groups receiving a regular diet or a diet supplemented with oyster shell powder (OSPow), oyster shell calcium (OSCa), or oyster shell extract (OSEx). After 16 weeks, tongue tissue samples from the mice were isolated for histological evaluation. The results showed a significant reduction in the incidence of tongue tumors in the groups receiving OSPow and OSCa. The underlying mechanism may involve calcium from the oysters, which significantly reduced the levels of proliferating cell nuclear antigen (PCNA) in the normal oral epithelium of the mice, while significantly increasing the levels of keratin 1, involucrin, filaggrin, and loricrin.^59^

### Wound healing properties

Wound healing is a continuous and complex repair process. Recent research has demonstrated that mollusk shells contribute positively to wound healing, either directly or indirectly, through mechanisms including hemostasis, epithelial repair, anti-inflammatory effects, and antibacterial action.^79^

Abalone (*Haliotis diversicolor*) shell has shown efficacy in accelerating the healing of burn wounds by promoting tissue remodeling, enhancing immune responses, and reducing inflammation. *In vitro* studies have demonstrated that abalone shell solutions stimulate macrophage proliferation while inhibiting inflammation caused by LPS through the downregulation of iNOS expression. *In vivo* studies further support these findings, showing that abalone shell treatment enhances the expression of transforming growth factor β 1 (TGF-β1) and increases type I collagen content in burn-injured rats, promoting faster recovery and tissue regeneration.^60^ Water-soluble components of pearl oyster (*Pinctada martensii*) shell (WSN) contribute to wound healing by promoting angiogenesis. WSN stimulates the proliferation of murine fibroblast NIH3T3cells and type I collagen synthesis, enhances collagen deposition at burn sites, accelerates necrotic tissue clearance, and promotes granulation tissue formation.^61^

Cuttlebone has hemostatic and anti-inflammatory effects. The aqueous extract of cuttlebone shortens prothrombin time (PT) and activated partial thromboplastin time (APTT) in mouse plasma, with a dose-dependent procoagulant effect observed *in vitro*.^62^ Additionally, polysaccharides derived from cuttlebone (*Sepia esculenta Hoyle*) reduce inflammation by lowering serum levels of inflammatory cytokines, such as interleukin-6 (IL-6) and tumor necrosis factor-alpha (TNF-α), thereby accelerating the healing of oral ulcers.^80^

### Gastrointestinal protective properties

The gastrointestinal mucosa covers the entire gastrointestinal tract and plays a vital physiological role. Damage to the mucosa and disruption of the mucosal barrier can lead to various gastrointestinal diseases, such as ulcers and gastritis. MMSs and their derivatives have demonstrated protective effects in maintaining the integrity of the gastrointestinal mucosa, reducing inflammation, and mitigating gastrointestinal injury.

Research has shown that aqueous extracts of MMSs and calcined shells possess significant protective effects against gastric ulcers in rat models induced by anhydrous ethanol and nonsteroidal anti-inflammatory drugs (NSAIDs). The anti-ulcer activity is positively correlated with dosage.^63^ These extracts suppress gastric fluid secretion, lower gastric acidity, decrease pepsin activity, and significantly reduce the ulcer index, thereby alleviating mucosal damage.^64^ Additionally, they stimulate antioxidant factors, increasing NO and prostaglandin E2 (PGE2) levels in gastric tissue, as well as SOD and epidermal growth factor (EGF) levels in serum, while lowering serum MDA levels.^65^^,^^81^

Furthermore, cuttlebone (*Sepiella maindroni de Rochebrune*) polysaccharide CPS-1 has been shown to improve intestinal mucosal ulcers induced by Dextran Sulfate Sodium (DSS) in mice, accelerating the healing and repair of ulcerated tissues. It significantly increases the expression of EGF and platelet-derived growth factor (PDGF) in the blood, while lowering TNF-α levels, thus alleviating ulcerative colitis.^66^ When compared to existing synthetic drugs such as Omeprazole and Ranitidine, natural compounds derived from MMSs demonstrate comparable efficacy with fewer side effects, making them more suitable for the long-term treatment of gastrointestinal diseases. Thus, MMSs hold promise as a safer and more effective alternative for the long-term management of gastrointestinal conditions.

### Hepatoprotective properties

MMSs have demonstrated significant hepatoprotective properties, playing a crucial role in safeguarding liver function and preventing liver damage. Non-alcoholic fatty liver disease (NAFLD), one of the most common liver diseases worldwide, is characterized by lipid accumulation. Ethanol extracts from oyster (*Crassostrea gigas*) shell have shown lipid-lowering effects, specifically reducing triglyceride (TG) levels in murine Hepa1c1c7 hepatoma cells. This lipid reduction is achieved initially through the inhibition of serine palmitoyl transferase (SPT) subunits Sptlc1 and Sptlc2, which diminishes sphinganine (SPA) and sphingomyelin (SM) synthesis. Subsequently, the expression of key lipogenic genes, including acetyl CoA carboxylase (ACC), fatty acid synthase (FAS), stearoyl CoA desaturase-1 (SCD1), and diacylglycerol acyltransferase 2 (DGAT2), is downregulated, further suppressing lipid synthesis.^67^

In the context of acute liver injury induced by carbon tetrachloride (CCl_4_), both the aqueous extracts of abalone (*Haliotis discus hannai Ino*, *Haliotis ruber*, *Haliotis laevigata*) shells and chitosan derived from cuttlebone (*Sepia kobiensis*) exhibit potent liver-protective effects. The aqueous extract of abalone shell increases liver glycogen content, alleviates inflammation in the portal area, and promotes the regeneration of hepatocytes in mice.^68^ Simultaneously, chitosan from cuttlebone significantly decreases hepatic MDA levels in rats, while elevating non-enzymatic antioxidants such as glutathione (GSH), and enhancing antioxidant enzyme activities, including superoxide dismutase (SOD), catalase (CAT), and glutathione peroxidase (GPx). This intervention also leads to reductions in plasma and tissue levels of alanine transaminase (ALT) and aspartate aminotransferase (AST), which are key indicators of liver damage. Additionally, chitosan reduces levels of free fatty acids (FFAs), triglycerides, total cholesterol (TC), and high-density lipoprotein cholesterol (HDL-C), further underscoring its hepatoprotective potential.^69^

### Neuroprotective activity

Among MMSs, pearl oyster (*Pinctada’*s pearl oyster family) shells demonstrate significant neuroprotective effects, including the restoration of memory impairments, the alleviation of depression and anxiety, and sedative-hypnotic properties.

When LPS is administered either centrally or peripherally in rats and mice models, it induces symptoms such as reduced activity, anxiety, depression, and cognitive dysfunction. Pearl oyster (*Pinctada maxima*) shell extracts can restore the expression of 5-hydroxytryptamine (HT)1A receptors, which are decreased by LPS, and the expression of 5-HT2A receptors, which are increased by LPS, by enhancing the expression of nuclear factor E2-related factor (Nrf2). Furthermore, it restores the expression levels of brain-derived neurotrophic factor (BDNF).^70^

Sulfated polysaccharides isolated from pearl oyster (*Pinctada fucata*) shell have also been shown to reverse scopolamine-induced memory impairment in mice. This neuroprotective effect is primarily linked to the reduction of oxidative stress through decreased MDA levels and enhanced activity of copper-zinc superoxide dismutase (Cu, Zn-SOD). Furthermore, these polysaccharides suppress the overexpression of pro-inflammatory cytokines such as IL-1β, IL-6, and TNF-α, while promoting the expression of BDNF and nerve growth factor (NGF) through the increased phosphorylation of extracellular signal-regulated kinase (ERK) and cAMP response element-binding protein (CREB).^71^

Moreover, pearl oyster shell powder, water-soluble proteins, and conchiolin proteins have been shown to exert sedative-hypnotic effects. These compounds significantly prolong the latency period before seizures in pentylenetetrazol (PTZ)-induced seizure models in mice, modulating neurotransmitter activity by downregulating 5-HT3 expression and upregulating gamma-aminobutyric acid (GABA) levels in brain tissue.^72^

Although the biological activities of MMSs are well documented across *in vitro* and *in vivo* models (Table 1), current research remains limited in several important aspects. Most existing studies rely on crude extracts or partially characterized components, making it difficult to attribute specific bioactivities to defined molecular structures. Moreover, interspecies variability in shell composition and the lack of standardized extraction and testing protocols hinder cross-study comparisons and mechanistic understanding. Critically, clinical evidence supporting the therapeutic benefits of MMS-derived components is still scarce, restricting their translation into functional foods or biomedical products.

To advance the field, future studies should focus on isolating and characterizing specific active constituents-including matrix proteins, peptides, and polysaccharides-and elucidating their mechanisms using omics, structural biology, and high-throughput screening approaches. More robust animal models and well-designed clinical studies are also needed to validate efficacy and safety. Establishing standardized bioactivity evaluation frameworks will be essential for transforming MMSs from traditional remedies into scientifically supported bioactive materials.

## Applications of marine mollusk shells

**Figure 4:**
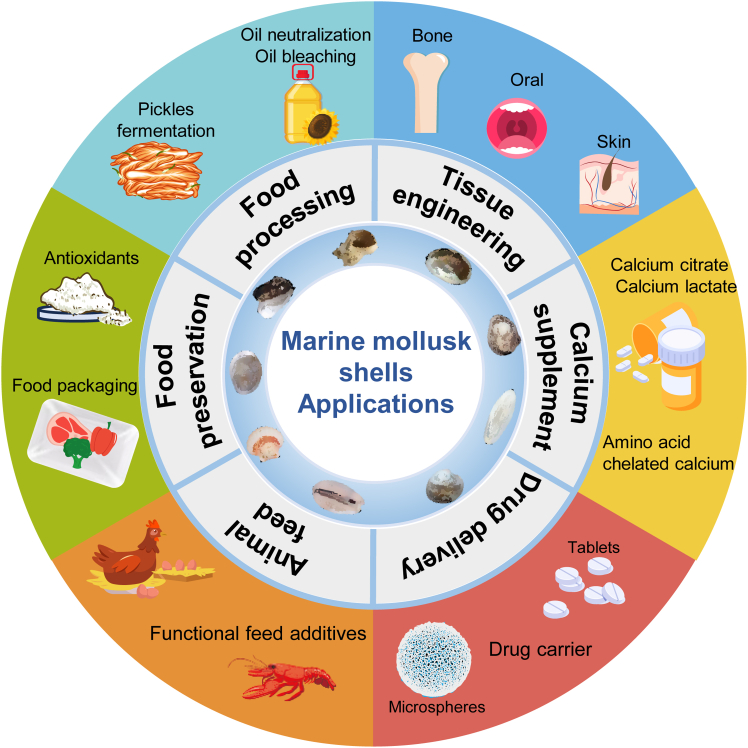
Applications of marine mollusk shells (MMSs)

MMSs and their derivatives, owing to their unique structure and bioactive compounds with health-promoting effects, are regarded as promising and sustainable resources. Currently, MMSs are being applied in various fields, including animal feed, food processing, food preservation, and calcium supplements, as well as in tissue engineering and drug delivery systems (Figure 4).

### Feed and AquaFeed

MMSs offer significant potential as functional feed additives for both poultry and aquatic animals, contributing to improved animal growth performance, strengthened immunity, and enhanced quality of animal products.

Adding 6% MMSs powder to the feed of Australian red claw crayfish (Cherax quadricarinatus) sufficiently fulfills the calcium requirements for optimal crayfish growth, increases calcium content in the exoskeleton, ensures shell hardening, facilitates faster molting, and improves survival rates. Compared with conventional calcium carbonate, natural shell calcium exhibits higher bioavailability.^82^ Furthermore, Scallop shell can be processed into active dicalcium phosphate (ADP), adding 1.05% ADP to the diet of broiler chickens effectively promotes nutrient absorption, enhances carcass traits and meat quality, while also playing a critical role in maintaining a healthy gut microbiota, improving intestinal development, and optimizing the microbial environment.^83^ In the case of laying hens, supplementing feed with MMS powder has been proven to boost egg production, increase egg weight, and improve eggshell strength, thereby enhancing both the quality and nutritional value of the eggs.^84^

### Food processing

Recent studies have demonstrated that MMSs and their derivatives play important roles in the pickle fermentation process as well as in the refining of vegetable oils.

Pickles are a traditional fermented vegetable product, and controlling lactic acid bacteria remains a critical issue in pickle production. Calcium extracts from oyster shell have been shown to inhibit the formation of excessive acidic substances during fermentation, thus preventing over-acidification. while maintaining the activity of lactic acid bacteria and preserving the natural flavor of the pickles.^85^

MMSs can be thermally decomposed to produce nearly pure CaO, a mild alkali that serves as an effective neutralizing agent for sunflower oil. When used, it removes approximately 61% of free acids, significantly reducing the oil’s acidity without causing any oil loss. Compared to traditional neutralizers such as NaOH, CaO derived from MMSs preserves the oil’s quality by preventing sterol degradation, reducing totox values, and minimizing oxidation byproducts.^86^ Moreover, their porous structure and surface chemical properties of MMS powder allow them to effectively adsorb pigments, impurities, and chromogenic substances, which leads to a visible bleaching outcome in the bleaching process of sunflower oil. It substantially reduces pigments such as chlorophyll and carotenoid and demonstrates superior performance in eliminating peroxides.^87^

### Food preservation

Food spoilage and the growth of pathogenic microorganisms are major contributors to food deterioration and foodborne illnesses, posing significant challenges to the food industry. Effective preservation technologies are essential to address these issues. As a natural material, MMSs offer promising potential in food preservation by inhibiting microbial growth, preventing foodborne diseases, extending shelf life, and ensuring food safety.

Calcined scallop shell powder (HSSP) has been shown to serve as an effective antibacterial alternative, providing thorough coverage to vegetables and meat through the impregnation method, thus meeting food preservation requirements. When applied at a 5% concentration, HSSP significantly reduces the total aerobic bacterial count and coliform bacteria in chicken meat, while also effectively inhibiting the foodborne pathogen Listeria monocytogenes.^88^ Sorbitol-coated HSSP (SC-HSSP) enhances moisture absorption while inhibiting the formation of CaCO_3_ scale, without compromising its antibacterial efficacy. SC-HSSP can be used in the treatment of fresh vegetables, such as lettuce and cucumbers, offering an effective method for microbial control.^89^

In addition to direct applications in food preservation, MMS powders can be incorporated into polymer matrices for the development of food packaging materials. For example, composite materials made from HSSP and modified polylactide (MPLA) demonstrate excellent compatibility, robust antibacterial activity, and biodegradability.^90^ Furthermore, scallop shell powder, after wet modification, can be blended with chitosan through tape casting to produce scallop shell powder/chitosan composite films. These films exhibit superior mechanical properties, transparency, and low water vapor permeability, making them ideal for fruit preservation by reducing respiration, minimizing moisture loss, and extending shelf life.^91^

### Calcium supplement

Calcium is essential for human health. In recent years, with the growing awareness of calcium supplementation worldwide, the demand for calcium supplements has been increasing. Conventional inorganic calcium, such as calcium carbonate, requires dissociation into calcium ions by gastric acid before it can be absorbed. However, its low solubility, poor absorption, and potential to cause gastrointestinal side effects limit its effectiveness. In contrast, calcium derived from MMSs exhibits high safety and bioavailability, which can serve as a natural source of organic calcium and often is a promising candidate for developing calcium supplements.

MMSs can be processed to produce organic calcium compounds, such as calcium citrate,^92^ calcium lactate,^93^ and amino acid chelated calcium,^94^ through methods such as high-temperature calcination, substitution, precipitation, and water system synthesis. These organic calcium compounds exhibit a variety of microscopic morphologies and contain essential trace elements such as iron, zinc, and copper, which are vital for human health. They also demonstrate high solubility and bioactivity. Abalone shell, as a calcium source, can undergo water system synthesis with composite amino acids (Glycine: Threonine = 1:3) to produce composite amino acid chelated calcium. This chelated calcium supplement not only fulfills the body’s calcium requirements but also provides the dual benefit of supplementing amino acids.^94^ Additionally, L-aspartic acid chelated calcium synthesized (ACOS) from oyster (*Crassostrea gigas*) shell through the water system method has been shown to reverse bone calcium loss in osteoporotic rats, alleviating calcium deficiency. ACOS has demonstrated efficacy in restoring bone tissue structure, improving osteoporosis, and has a high safety profile with no significant toxic side effects.^95^

These findings provide strong scientific support for developing MMS-based calcium supplements as safe and effective alternatives to conventional options.

### Tissue engineering

Tissue engineering, a critical branch of regenerative medicine, has become a well-established approach for reconstructing damaged, injured, or missing tissues and organs. The fundamental concept involves seeding functionally relevant living cells onto porous scaffolds to create biological substitutes, which are then transplanted into damaged tissue sites to repair injuries and restore organ function, enabling the regeneration of tissues or organs.^96^ Due to their excellent mechanical properties and biocompatibility, MMSs have garnered attention as natural biomaterials, particularly in bone, oral, and skin tissue engineering applications.

In bone tissue engineering, MMSs offer promising potential both in enhancing existing bone repair materials and in developing novel scaffolds. Combining abalone shell with calcium sulfate through a condensation cycle, constant temperature oil bath heating method yields injectable bone cement, which is suitable for reconstructing irregular bone defects. This bone cement has been demonstrated to promote osteoblast proliferation and differentiation in rat models with bone defects, enabling endogenous bone regeneration.^97^ Similarly, the incorporation of abalone shell powder into polycaprolactone (PCL) via 3D printing technology produces Aba/PCL composite scaffolds with favorable mechanical stability and biocompatibility. These scaffolds facilitate osteoblast migration, upregulate genes related to osteogenesis, and aid in repairing cranial defects in rats.^98^ Furthermore, scaffolds composed of oyster shell and alpha-calcium sulfate hemihydrate (α-CSH) have shown efficacy in cranial repair. When these scaffolds are combined with platelet-rich plasma (PRP) and bone mesenchymal stem cells (BMSCs), they provide a targeted, cell-loaded therapeutic strategy that significantly improves the healing of critical-size calvarial defects.^99^

In oral tissue engineering, MMSs hold great promise for applications in dental, periodontal, and dental implant treatments. Abalone shell-derived calcium carbonate (Anadara granosa) shows potential as a dentin-pulp material for restorative therapies. In a mouse model of mechanical dentin injury, abalone shell calcium carbonate has been found to suppress NF-kB expression while increasing the expression of TGF-β1 and VEGF-A in pulp tissue, demonstrating bioactive properties conducive to dentin regeneration.^100^ Additionally, the pearl oyster shell/PU/POSS composite, composed of pearl oyster shell, polyurethane (PU), and polyhedral oligomeric silsesquioxane (POSS), has been used to create a 3D porous scaffold for alveolar ridge preservation. When grafted into the sockets of rats following mandibular incisor extraction, this scaffold significantly reduces alveolar ridge resorption while promoting bone formation and mineralization.^101^ In dental implantation, combining pearl oyster shell powder with surgical implants stimulates bone formation around the implant surface, promoting peri-implant osteogenesis.^102^

For skin tissue engineering, cuttlebone-derived HAP combined with reduced graphene oxide (rGO) has been incorporated into a carrageenan biopolymer matrix to form the CAR-rGO-CF-HAp membrane. This bioactive membrane has demonstrated antibacterial, anti-inflammatory, and excellent wound healing properties in a zebrafish burn model, making it a strong candidate for wound reconstruction materials.^103^ In addition, Gel/Cb electrospun membranes, fabricated by electrospinning cuttlebone with gelatin, offer an effective dressing for tissue engineering. The incorporation of cuttlebone significantly reduces the hemolysis rate of the electrospun membranes. In a mouse tail break test, Gel/Cb treatment accelerated coagulation, leading to a significant reduction in bleeding time and volume, indicating strong hemostatic properties.^104^

### Drug delivery

MMSs possess a natural porous structure, with large, evenly distributed pores. Through processes such as crushing and calcination, these pores can be modified to create functional structures with enhanced adsorption properties, making them highly suitable for drug delivery systems.

Superfine grinding significantly increases the surface area and porosity of oyster shell, resulting in excellent solubility, dispersibility, and adsorption capacity. When combined with Sodium Carboxymethyl Cellulose (CMC) as a composite drug carrier, floating aspirin (ASP) tablets have been developed. This formulation effectively reduces the “burst release effect,” extends gastric retention time, maintains steady drug levels in the blood, improves absorption, and enhances therapeutic efficacy.^105^ In addition, ultrafine oyster shell powder, modified with soluble starch, demonstrates uniform granulation, improved dispersibility, and superior encapsulation properties. This powder, used in a solution method, can form antioxidant inclusion complexes with vitamin C (VC) and vitamin E (VE), showing stable, sustained-release effects and enhancing the antioxidant properties of the formulation.^106^

Moreover, MMS-based carriers can control drug release and prolong its duration in acidic tumor microenvironments. Spherical calcium carbonate nanoparticles (CSCaCO_3_NP) synthesized from ark (*Anadara granosa*) shell using Tween 80 and mechanical dry ball milling have a high drug-loading capacity. By loading gefitinib (GEF) and paclitaxel (PTXL) onto CSCaCO_3_NP, dual drug-loaded GEF-PTXL-CSCaCO_3_NP was produced, which demonstrates excellent alkalization properties and enables slow, sustained drug release in moderately acidic pH environments.^107^ Abalone shell, utilized as a calcium source, can be synthesized into porous bio-hydroxyapatite (bio-HAP) microspheres via hydrothermal methods. These microspheres exhibit excellent biocompatibility and pH responsiveness. DOX-HAP microspheres, loaded with doxorubicin (DOX), achieve high encapsulation efficiency (95.542%) and effectively induce apoptosis in MCF-7 cells, with apoptosis rates rising as the HAP concentration in the DOX-HAP microspheres increases.^108^

## Safety of mollusk shells

Ensuring the safety of MMSs for food, nutraceutical, and medicinal applications requires comprehensive control of potential contaminants—particularly heavy metals and microplastics. These risks stem primarily from environmental pollution and bioaccumulation in aquatic organisms, underscoring the importance of source control, standardized monitoring, and validated decontamination processes.

### Heavy metals

Heavy metals such as arsenic, cadmium, lead, chromium, and copper are common aquatic pollutants known for their nephrotoxicity, hepatotoxicity, neurotoxicity, and adverse effects on the cardiovascular and immune systems.^109^ Mollusks living in aquatic environments tend to accumulate these metals in both their soft tissues and shells. The presence of heavy metals in mollusk shells can compromise the safety and effectiveness of MMS-derived materials and poses potential health risks to humans. Therefore, the monitoring and control of heavy-metal levels in MMSs are crucial for ensuring their safe utilization.^5^

Regular monitoring of water quality in the habitats where wild or farmed mollusks are sourced represents the first line of safety control, helping to ensure low levels of environmental contamination.^110^ In parallel, the direct analysis of MMSs is essential, and recent advances in analytical technologies have significantly improved the efficiency and accuracy of heavy-metal quantification. Inductively coupled plasma techniques—ICP-mass spectrometry (ICP-MS) and ICP-atomic emission spectroscopy (ICP-AES)—are among the most widely used and reliable methods. Emerging approaches such as laser-induced breakdown spectroscopy (LIBS) and X-ray fluorescence spectroscopy (XRFS) further strengthen the capacity for rapid and non-destructive detection, enhancing quality control for MMS applications.^109^

The 2025 edition of *the Pharmacopoeia of the People’s Republic of China* specifies strict quantitative limits for heavy metals and toxic elements in oyster shells, requiring Pb ≤ 5 mg/kg, Cd ≤ 0.3 mg/kg, As ≤ 2 mg/kg, Hg ≤ 0.2 mg/kg, and Cu ≤ 20 mg/kg, determined using atomic absorption spectroscopy or ICP-MS.^10^ However, no unified international regulatory standards currently exist for heavy-metal control in MMS-derived materials intended for biomedical use. To ensure safety and promote global harmonization, future efforts should establish systematic risk-assessment frameworks and develop internationally applicable upper limits and standardized testing protocols.

In addition to monitoring, certain processing technologies—such as thermal treatment and calcination—can reduce some heavy-metal burdens and simultaneously increase the proportion of major mineral components, thereby improving the safety profile of MMSs. Recent analyses of 22 batches of oyster shells before and after calcination demonstrated significant decreases in heavy-metal content: Cu decreased from an average of 1.3305 mg kg^−1^ to 0.2975 mg kg^−1^, As from 0.2426 mg kg^−1^ to 0.0826 mg kg^−1^, Hg from 0.0816 mg kg^−1^ to 0.0113 mg kg^−1^, and Pb from 1.3485 mg kg^−1^ to 0.2613 mg kg^−1^. These reductions confirm that calcination can effectively lower residual heavy metals while simultaneously purifying the mineral fraction of the shell.^111^ Nonetheless, raw-material screening and environmental control remain essential, as intrinsic heavy-metal deposition cannot be eliminated through post-processing alone.

### Microplastics

Microplastics have emerged as a major class of contaminants in marine organisms, and filter-feeding mollusks are among the species most susceptible to their ingestion.^112^ Studies indicate that microplastic accumulation within biological tissues is largely irreversible, and human exposure may adversely affect growth, development, and metabolic functions.^113^ However, standardized protocols for characterizing, analyzing, and detecting microplastics are still incomplete, and toxicological evidence remains limited, hindering comprehensive risk assessment.

Microplastics have been widely detected in common MMSs, yet their reliable detection and effective removal remain technically challenging. At present, no validated method exists to eliminate microplastics once they have been ingested by living organisms, making upstream mitigation the most feasible approach. Practical strategies include.

1. Source control—prioritizing shells obtained from certified clean-water aquaculture zones and favoring farmed over wild mollusks to reduce environmental exposure;
2. Enhanced removal during water treatment—applying natural organic flocculants such as chitosan to strengthen traditional coagulants (e.g., polyaluminum chloride), which has been shown to significantly increase microplastic removal efficiency;^114^
3. Routine environmental monitoring using FTIR- or Raman-based microplastic identification techniques to ensure continuous surveillance of aquaculture and harvesting environments.^115^

Together, these strategies help minimize microplastic exposure during shell formation and effectively reduce contamination risks in MMS-derived products.

## Conclusion

In conclusion, MMSs represent an abundant yet underutilized biomaterial resource with substantial potential for value-added applications. Their unique hierarchical structures and diverse bioactive constituents—including minerals, proteins, and chitin—form a natural basis for multifunctional applications. Rapid progress in extraction and processing technologies has expanded the range of recoverable functional components, enabling their exploration in food science and biomedicine. Accumulating evidence further demonstrates that MMS-derived materials possess extensive biological activities, such as antioxidant, antimicrobial, anti-osteoporotic, antihypertensive, anti-inflammatory, immunomodulatory, and antitumor properties, highlighting their promise for functional foods and health-promoting products. Nevertheless, concerns regarding heavy metal accumulation, microplastic contamination, and species-dependent compositional variability underscore the need for rigorous safety evaluation and standardized processing frameworks to support industrial-scale translation.

Despite the advantages of MMSs demonstrated by the studies reviewed herein, several limitations reflect the current state of global MMS research.

### Limited species coverage and uneven evidence distribution

Although the applications summarized in this review are derived from different marine mollusk taxa, the current literature is unevenly distributed among species. Most existing studies focus on a narrow subset of species (e.g., oysters, mussels, scallops, and abalone), while many mollusks remain unexamined. This restricts the ability to draw cross-species comparisons regarding extraction efficiency, matrix composition, or biological activities. Accordingly, future research should give priority to standardized, cross-taxa studies aimed at uncovering the functional properties and utilization value of MMs.

### Geographic and disciplinary imbalance in research output

Pharmacological studies are primarily concentrated in China due to traditional medicinal usage, whereas research outside China has focused more on structural analysis or materials engineering. Consequently, globally validated mechanistic insights—especially for proteins, peptides, and matrix components—remain limited.

### Industrial bottleneck in recovery technologies

Despite the development of various methods for recovering functional components from MMSs, most techniques are still limited to laboratory settings and have not been scaled for industrial food production. Existing extraction methods, particularly for organic matrices with low contents, suffer from low yields and can damage the inorganic matrix during demineralization. Therefore, there is a pressing need to develop new technologies that can efficiently recover both organic and inorganic components simultaneously, thereby maximizing the overall utilization of MMSs.

### Insufficient toxicological and safety assessment

Quantitative evaluation of contaminants (e.g., heavy metals and environmental pollutants) remains inconsistent across studies. Standardized risk assessment, purity assurance, and quality-control frameworks are still lacking, limiting industrial or clinical translation.

### Limited translational and clinical evidence

Although promising results have been reported *in vitro* and in small animal models, validated preclinical and clinical evidence is nearly absent. To realize the therapeutic potential of MMSs, systematic research on extraction standardization, component characterization, efficacy, and safety testing is urgently required.

## Acknowledgments

This work was supported by the 10.13039/501100007129Shandong Provincial Natural Science Foundation (No. ZR2024MD105 and ZR2022LZY026); the NATCM’s Project of High-level Construction of Key TCM Disciplines (Marine Traditional Chinese Medicine; No. zyyzdxk-2023124); the Taishan Scholars Program (NO. tstp20240825); the Shandong Province Key Discipline Construction Project of Traditional Chinese Medicine (Marine Traditional Chinese Medicine); and the Youth Innovation Team of Higher Education Institutions in Shandong (No. 2022KJ256).

## Author contributions

J.-Y.H.: writing – original draft, resources, methodology, and data curation. X.R.: writing – review and editing, writing – original draft, methodology, data curation, and conceptualization. X.-J.F.: writing – review and editing, writing – original draft, methodology, data curation, and conceptualization.

## Declaration of interests

The authors declare that they have no known competing financial interests or personal relationships that could have appeared to influence the work reported in this article.
